# Locally relapsed and metastatic uterine leiomyoma: A case report

**DOI:** 10.1186/1752-1947-2-308

**Published:** 2008-09-23

**Authors:** Ambrogio P Londero, Patrizia Perego, Costantino Mangioni, Ralph J Lellé, Franco Londero, Diego Marchesoni

**Affiliations:** 1 Clinica di Ginecologia ed Ostetricia, University of Udine, piazzale SM della Misericordia, Udine, Italy; 2 Istituto di Anatomia Patologica, University of Milano Bicocca, Milan, Italy; 3 Clinica di Ginecologia ed Ostetricia, University of Milano, via Mayr, Milan, Italy; 4 Klinik und Poliklinik für Frauenheilkunde und Geburtshilfe, Albert-Schweitzer-Straße, Münster, Germany; 5 UO Ostetricia e Ginecologia – San Polo, via Galvani, Monfalcone, Italy

## Abstract

**Introduction:**

Benign metastasising leiomyoma refers to a type of lesion characterised by leiomyomatous alterations without any indication of malignancy. It presents as either a singular nodule or multiple nodules of proliferating smooth muscle cells and is generally found in the lungs of women who have undergone a hysterectomy. The purpose of this case report is to contribute to the knowledge of this rare disease by presenting evidence and experience of a patient case. In particular, this report seeks to investigate the therapeutic approaches in order to understand whether a standard of care can be prescribed and whether the use of prophylaxis therapy with progesterone as a follow-up to surgery serves as a reasonable treatment in certain cases diagnosed as benign metastasising leiomyoma.

**Case presentation:**

We present the case of a 52-year-old Caucasian woman who developed a pelvic relapse and a pulmonary localisation of benign metastasising leiomyoma following a hysterectomy for myomatous uterus.

**Conclusion:**

Our literature review revealed a single case of the use of chemoprophylaxis as treatment of a benign metastasising leiomyoma. The role of chemoprophylaxis in preventing future recurrences remains unclear. The use of progesterone as an adjuvant therapy for benign metastasising leiomyoma could simply be palliative, with associated psychological benefits, or it could be of therapeutic significance.

## Introduction

There are conditions, although rare, in which histologically apparently benign leiomyomas of corpus uteri extend beyond their usual boundaries or are associated with extra-uterine leiomyomas. The term benign metastasising leiomyoma (BML) refers to a type of lesion characterised by leiomyomatous alterations without any indication of malignancy. It presents as single or multiple nodules of proliferating smooth muscle cells, usually in the lungs of women who have undergone a hysterectomy.

Controversy exists as to whether lung leiomyomas represent the synchronous or metachronous development of an independent lung lesion; the term more readily describes the metastases of a benign uterine primary tumour, and certainly recent molecular investigators tend to favour this latter definition [1].

The purpose of this report is to contribute to the knowledge of this rare disease by presenting evidence and our experience of a patient case. In particular, this case report seeks to investigate the therapeutic approaches in order to understand whether a standard of care can be prescribed and whether the use of prophylaxis therapy as a follow-up to surgery serves as a reasonable treatment in certain cases diagnosed as BML.

## Case presentation

A 52-year-old Caucasian woman, gravida IV, para 2, abortus 2, with a 5-year history of uterine leiomyomas, presented in January 1999 with a pelvic mass of 87 mm at its greatest diameter at sonography. In July 2000, the mass had increased in volume, and in January 2001, the patient was admitted to hospital for a total abdominal hysterectomy with a bilateral salpingo-oophorectomy owing to uterine leiomyomas.

At laparotomy, the uterus was found to be three times the normal size (14 cm × 14.5 cm). In addition, a mass of about 30 mm in diameter located in the right laterocervical region was resected; this second mass was softer and less resistant to indentation than the leiomyomas inside the uterus. The ovaries appeared to be regular. The histological exam confirmed the presence of multiple leiomyomas of the corpus uteri and a right laterocervical leiomyoma. All of the specimens considered were positive for oestrogen and progesterone receptors. At 2-month follow-up, there was no evidence of disease. More than 10 years before admission, two incomplete abortions had occurred in the first trimester of pregnancy and had been followed by uterine curettages.

After 3 years, the patient presented with abdominal bloating and occasional abdominal pain. A pelvic sonographic examination revealed a mass of 12 cm at its greatest point. In December 2004, magnetic resonance imaging was carried out. This revealed the pelvic mass to be solid and non-homogeneous, as evidenced by the presence of haemorrhagic areas, necrosis and vascular structures; the non-homogeneous nature was reinforced following the injection of contrast medium whereby non-homogeneous high enhancement became apparent. The mass was connected to the right pelvic wall via a vascular supply that appeared to originate from the iliac vessels in the obturator region. The rectum and bladder walls did not appear to be affected by the growth of the mass. A positron emission tomography-computed tomography (PET-CT) total body scan was performed, from which a pelvic localisation with low glucose affinity could be concluded. There were no pulmonary masses on pre-operative chest X-ray.

In order to remove the mass, a second abdominal intervention was performed, which revealed no evidence of tumour invasion in the pelvis or abdominal viscera. The mass measured 13 cm × 9 cm × 6 cm, was delimited by serosa, and when cut appeared to be myxoid and with oedematous areas inside.

**Figure 1:**
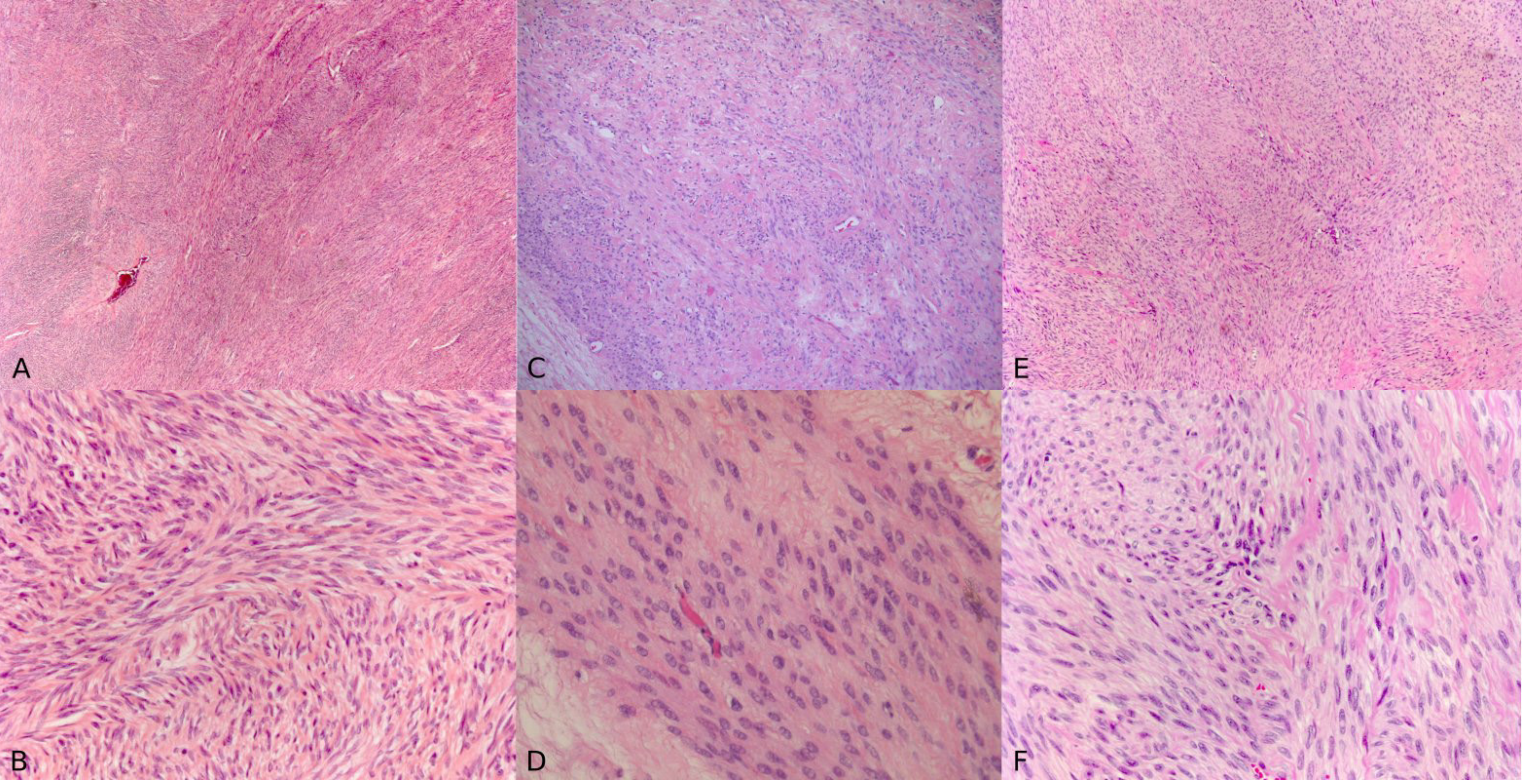
**(A), (B) Histological appearance of primary uterine leiomyoma.** (C), (D) Pelvic relapsed leiomyoma. (E), (F) Pulmonary mass (haematoxylin and eosin staining; (A), (C), (E) magnification ×10; (B), (D), (F) magnification ×40).

**Figure 2:**
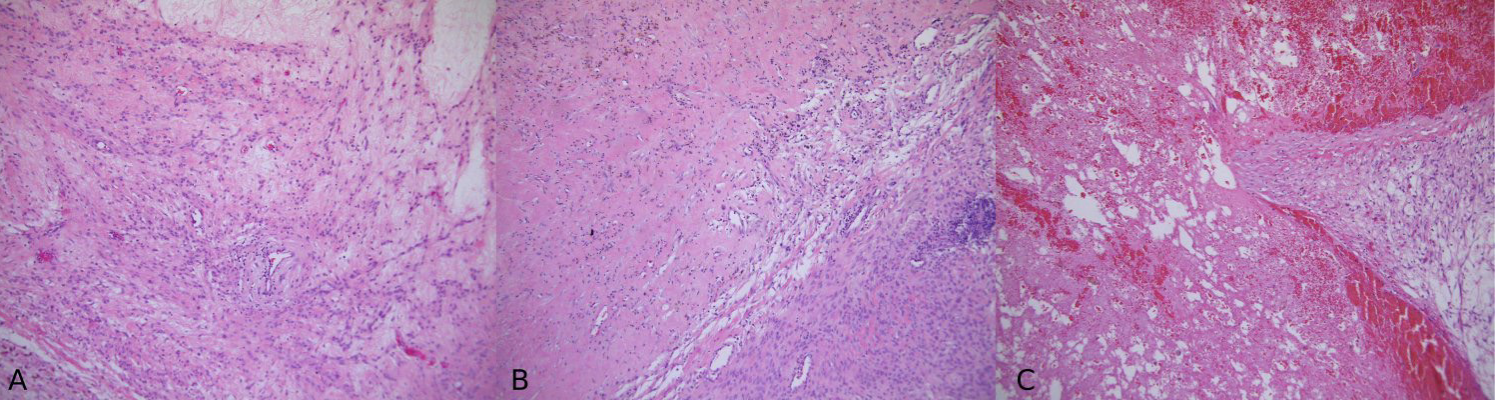
**(A) Area with oedematous aspect.** (B) Normal spindle-shaped smooth muscle cells and area of ischaemic necrosis with haemosiderin deposition. (C) Haemorrhagic area inside the mass (haematoxylin and eosin staining; magnification ×10).

**Figure 3:**
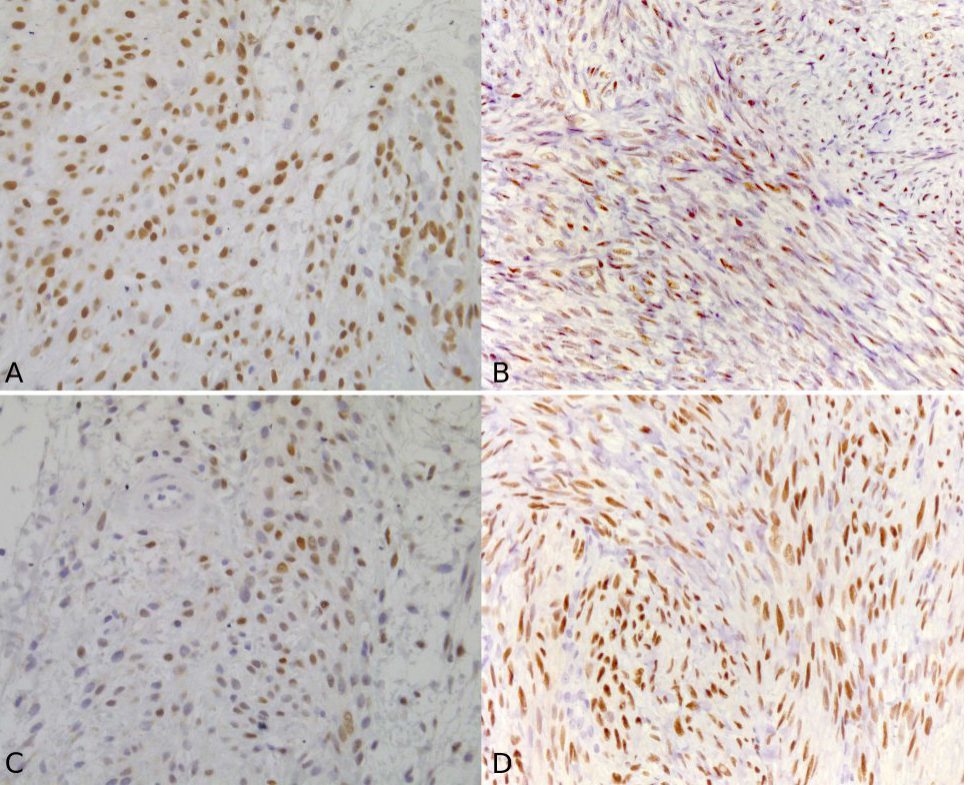
**(A) Immunohistochemical staining for oestrogen receptors of the pelvic relapsed mass.** (B) Immunohistochemical staining for oestrogen receptors of the pulmonary mass. (C) Immunohistochemical staining for progesterone receptors of the pelvic relapsed mass. (D) Immunohistochemical staining for progesterone receptors of the pulmonary mass (magnification ×40).

Histologically, spindle-shaped smooth muscle cells were present each containing a regular oval nucleus, without mitosis and with a moderate vascularisation and oedematous aspect. The cells were positive for smooth muscle specific actin, and there was a low proliferation index (2% of nucleus being MIB-1 positive). Again, the specimens expressed oestrogen and progesterone receptors (Figures 1 to 3). The final diagnosis was leiomyoma.

In light of the histological results and the lack of evidence to suggest immediate prescription of radiotherapy or chemotherapy, the decision was taken to withhold treatment and await observation at follow-up.

**Figure 4:**
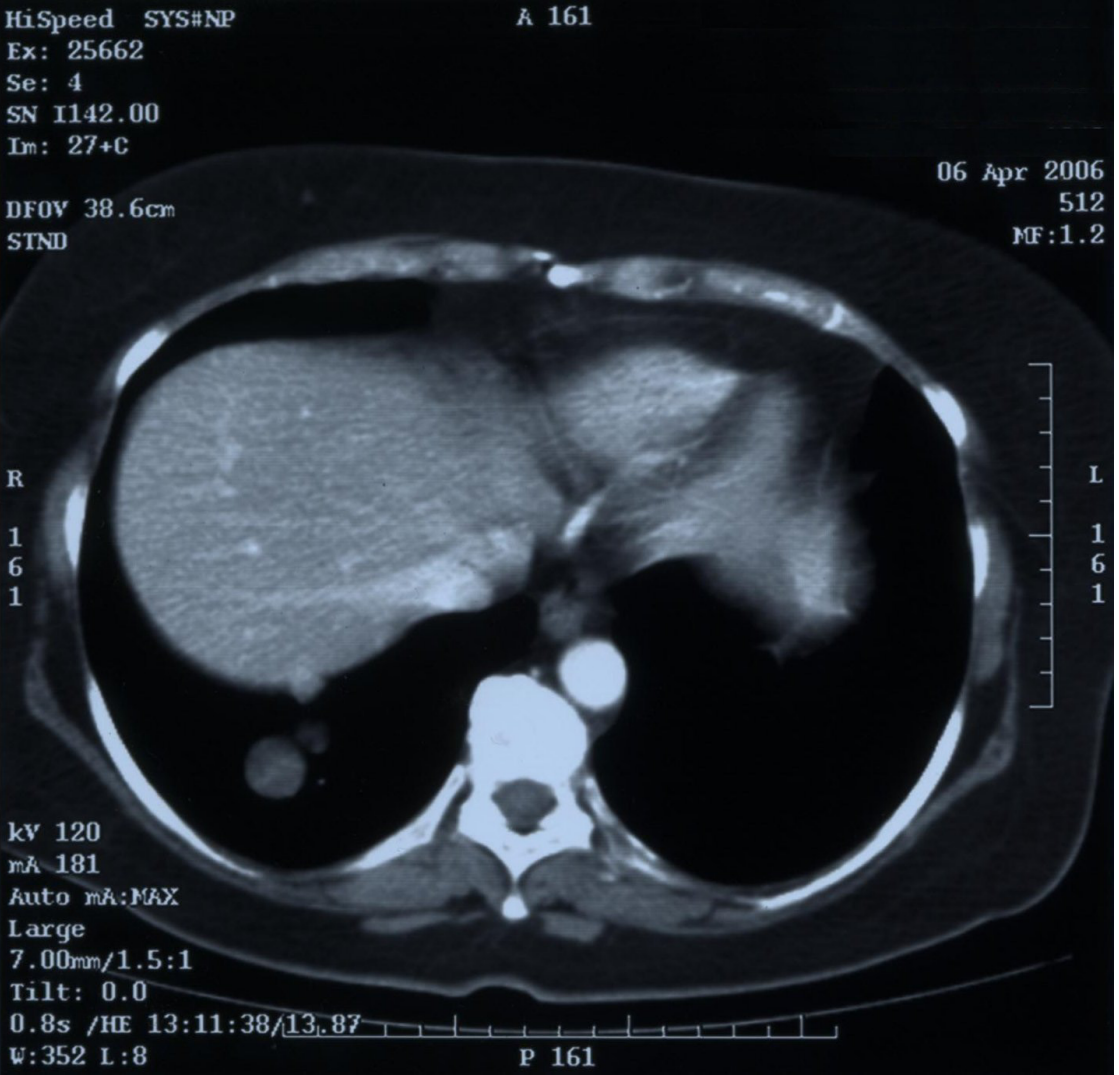
Computed tomography scan of the thorax showing single nodular mass in the right lung of about 4 cm in diameter, evident 1 year after the pelvic recurrence resection.

A routine chest X-ray the following year, in May 2005, showed a nodular posterior basal density in the right lung of about 4 cm in diameter. The presence of a single nodular mass was confirmed at CT scan of the thorax (Figure 4). At this point, a second PET-CT total body scan was performed, showing a pulmonary lesion with a low metabolic gradient, as is consistent with a benign lesion.

In June 2005, a right lower lung lobectomy was performed, during the course of which an intra-operative frozen section was also carried out. This failed to identify any malignant elements. A subsequent and more accurate histological examination permitted identification of the mass as a mesenchymal neoplasm with a smooth muscle differentiation, typifying a leiomyoma. Evidence to this effect was as follows: a low mitotic index of less than 1 mitosis per 10 high-power fields (HPFs); a low MIB-1 index of 1%; and a positive immunohistochemistry reaction for oestrogen receptors, progesterone receptors, H-caldesmon and desmins. Moreover, testing was negative for keratins, Bcl2, CD10 and CD99.

**Figure 5:**
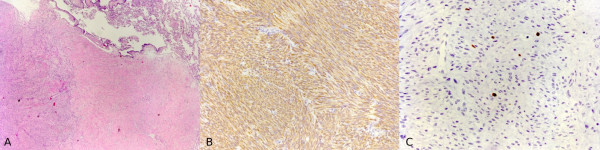
**(A) Histological appearance of the pulmonary mass with an area of fibrosis and an area that represents pulmonary tissue.** (B) Immunohistochemical staining of the pulmonary mass for desmin. (C) Immunohistochemical staining of the pulmonary mass for Ki-67.

A review of the histological pattern of the original mass and pulmonary mass showed low mitotic indices in both the pelvic mass (2 mitoses per 10 HPFs) and also the pulmonary mass (less than 1 mitosis per 10 HPFs) and that the histology of the masses was similar, typifying a leiomyoma (Figures 1, 3 and 5).

One month after the intervention, a course of chemotherapy prophylaxis was commenced using 80 mg per day of megestrol. At the 6-month follow-up, a CT scan of the thorax and abdomen was negative for masses; the 12-month examination was similarly negative.

## Discussion

In our literature review, we could only find around 100 documented cases that make reference to BML diagnoses [2]. Even if this is an under-diagnosed condition owing to the lack of symptoms, it is still a rare disease. The nodules in the lungs are usually detected post-hysterectomy, with detection ranging from some months up to decades.

In explaining the histogenesis of these lesions, opinion in the existing literature can be classified into three main streams. For proponents of the first approach, the uterine neoplasm is regarded as a low-grade leiomyosarcoma with malignant potential. The second approach centres on the presence of lung emboli formed of cells that originated from a benign leiomyoma of the uterus. The third and final hypothesis asserts that multi-focal smooth muscle proliferations may be the result of the independent growth of smooth muscle tissue in response to hormonal milieu.

Serving to validate the hypothesis that staminal mesenchymal cells from the myometrium are embolised to other organs (lung, lymph nodes), our patient, in common with many other documented cases, had a previous history of gynaecological surgery, including curettages and hysterectomy.

Lesions are discovered incidentally, although symptoms such as coughing, chest pain and dyspnoea have been noted. Furthermore, lesions produce differing outcomes, some of which lead ultimately to fatality.

The differential diagnosis for pulmonary nodules on chest radiography includes benign and malignant primary and metastatic neoplasms, vascular lesions, infectious and noninfectious inflammatory granuloma and collagen vascular disease. In every instance when a locally relapsed disease with a distant metastasis is discovered, all of the observed possibilities must be considered. One important step that brings us to diagnosis involves performing either a biopsy or a surgical resection.

Pulmonary specimens for histological analysis have been obtained through various methods, including percutaneous biopsy, transbronchial lung biopsy and lobectomy. The greatest challenge in distinguishing a leiomyoma from a leiomyosarcoma concerns assigning the intermediate combination of diagnostic criteria, in order to decide whether the patient is experiencing a benign or malignant disease.

When mesenchymal cell neoplasms are present, the histological differential diagnosis falls between BML, primary pulmonary leiomyoma, leiomyosarcoma, metastatic BML from a source other than female genital internal organs, hamartoma and lymphangioleiomyomatosis.

The first therapy proffered in the literature relates to the surgical resection of the pelvic recurrence and, where possible, the pulmonary localisations. Several authors noted a more favourable outcome after bilateral oophorectomy, including a curative aspect of this surgical intervention [3]. However, in some documented cases, as in ours, bilateral oophorectomy has no influence on the growing leiomyoma mass. Surgery aside, there is considerable discussion in the literature regarding drug treatment therapy for BML. In certain instances, the luteinising hormone-releasing hormone analogue (goserelin) had a therapeutic role where other hormonal therapies (medroxyprogesterone, tamoxifen) had failed to achieve the curative targets [4].

Tamoxifen has proven to be effective *in vitro *for decreasing cell numbers and for stopping cell proliferation [5], but its role remains uncertain *in vivo *[6]. Another selective oestrogen receptor modulator, raloxifene, was documented as a successful BML treatment when administered in association with an aromatase inhibitor [7]. Aromatase inhibitors represent a further drug category possessing the ability to reduce the volume of a leiomyoma [7]. Progesterone has proven to be effective in the treatment of BML metastasised to the lungs, and there are examples of a complete resolution after the administration of megestrol [2].

The utilisation of long-acting GnRH analogues, which suppress pituitary gonadotrophin biosynthesis by decreasing the number and sensitivity of GnRH receptors, has been documented with favourable results in several reports [8]. The literature documents the regression of metastatic lesions in certain instances. This can be attributed to the significant drop in oestrogen levels that occurs after the end of pregnancy [9] and after the surgical or physiological menopause [3].

A number of different classes of growth factors and apoptosis-related factors have been identified as having a high likelihood of affecting leiomyoma growth, vascularity and extracellular matrix deposition: epidermal growth factor, insulin-like growth factors, transforming growth factor-β family, platelet-derived growth factor, angiogenic factors, Bcl-2 protein, tumour necrosis factor-α and p53 protein.

The heterogeneity of leiomyoma growth within the same uterus, despite the identical exposure to circulating sex steroid concentrations, suggests the involvement of local growth factors. These are expressed differently in normal smooth muscle and leiomyoma, which indicates that such factors may be involved in paracrine stimulation [10].

These results indicate that future non-surgical treatments for leiomyomas may include compounds that block the actions of specific growth factors involved in the control of uterine smooth muscle cell proliferation and growth.

The complexity of the interactions existing between specific growth factors, hormonal composition and leiomyoma behaviour and development forms the basis for understanding how a certain therapy can achieve a positive outcome in one instance, and yet fail to take effect in others. In order to make an informed decision as to which treatment to administer, an improved characterisation of the molecular expression and genetics of leiomyoma is necessary. Such information will likely clarify the reasons why certain therapies appear more efficacious with specific types of leiomyoma [11]. Future research should further seek to identify markers of prognosis that are informative about the risk of developing a BML. Our evidence and experiences presented in this report tend to suggest that BML, rather than being a homogeneous classification, should be viewed as a more general term encompassing an inclusive range of leiomyoma displaying unique characteristics and thus following separate growth patterns. It is for precisely this reason that different outcomes are observed when a particular treatment is administered in separate cases.

A review of BML therapies was carried out with the intention of clarifying the rationale for the use of a drug prophylaxis to prevent recurrences. Following an analysis of the existing literature, it was ascertained that the use of progesterone (medroxyprogesteron) as a prophylactic agent was limited to a single documented case [12].

## Conclusion

In our patient, following two recurrences and consequent surgical resections within a short period, the decision was taken to use a chemoprophylaxis agent despite the uncertainty surrounding its role in preventing future recurrences [12]. As a concluding point, progesterone used as adjuvant therapy for BML could simply be palliative, with the associated psychological benefits, or it could be of therapeutic significance. Our conclusions are, however, limited owing to the scarcity of pathological cases investigating this theme.

## Abbreviations

BML: benign metastasising leiomyoma; CT: computed tomography; HPF: high-power field; MRI: magnetic resonance imaging; PET-CT: positron emission tomography-computed tomography.

## Competing interests

The authors declare that they have no competing interests.

## Authors' contributions

APL, RJL and DM carried out the literature research and drafted the manuscript. PP made pathology contributions. CM and FL carried out the clinical and surgical management and helped in drafting and the critical revision of the manuscript.

## Consent

Written informed consent was obtained from the patient for publication of this case report and any accompanying images. A copy of the written consent is available for review by the Editor-in-Chief of this journal.
